# Supplementary material for: Cryo-EM structures of the plant anion channel SLAC1 from Arabidopsis thaliana suggest a combined activation model
Source: Nat Commun. 2023 Nov 14;14:7345. doi: 10.1038/s41467-023-43193-3 (PMC10645844; doi:10.1038/s41467-023-43193-3)

Supplementary Information

Cryo-EM structures of the plant anion channel SLAC1 from *Arabidopsis thaliana* suggest a combined activation model

Yeongmok Lee[1], Hyeon Seong Jeong[2,3], Seoyeon Jung[1], Junmo Hwang[2], Chi Truc Han Le[1], Sung-Hoon Jun[4], Eun Jo Du[2], KyeongJin Kang[2], Beom-Gi Kim[5], Hyun-Ho Lim[2,3], and Sangho Lee[1,*]

[1]Department of Biological Sciences, Sungkyunkwan University, Suwon 16419, Republic of Korea

[2]Neurovascular Unit Research Group, Korea Brain Research Institute, Daegu 41068, Republic of Korea

[3]Department of Brain Sciences, Daegu Gyeongbuk Institute of Science & Technology (DGIST), Daegu 42988, Republic of Korea

[4]Electron Microscopy Research Center, Korea Basic Science Institute, Cheongju 28119, Republic of Korea

[5]Metabolic Engineering Division, National Institute of Agricultural Sciences, Rural Development Administration, Jeonju 54874, Republic of Korea

[*]Corresponding author: sangholee@skku.edu

Supplementary Figures 1 - 16

Supplementary Table 1

Supplementary References

Uncropped images of blots and gels

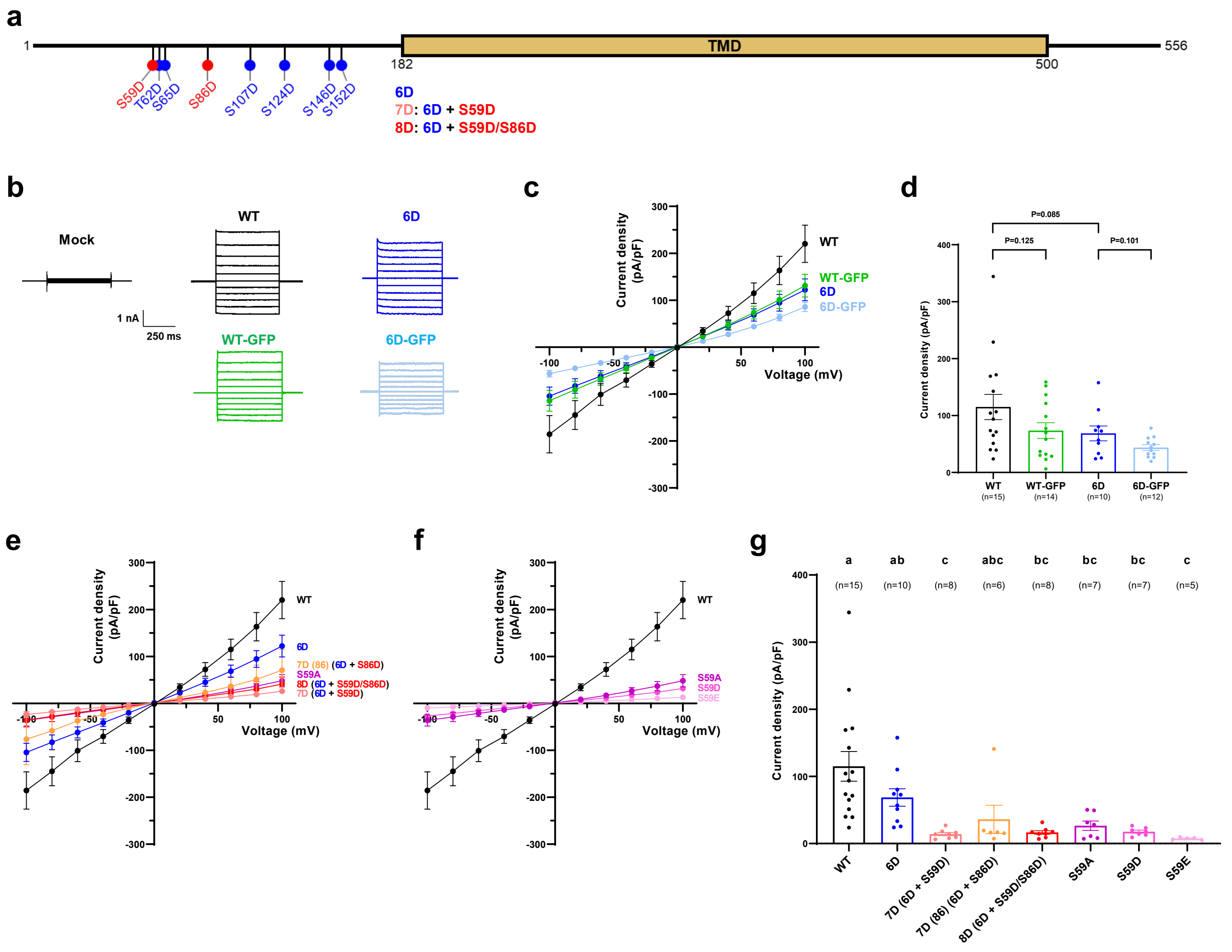

Supplementary Figure 1. Whole-cell patch-clamp recording of AtSLAC1 channels.

a, Domain architecture of AtSLAC1 showing phospho-mimetic mutations. Numbers indicate amino acid residue numbers. “6D” (blue) refers to T62D/S65D/S107D/S124D/S146D/S152D; “7D” (light red) the “6D” with S59D; “8D” (red) the “6D” with S59D/S86D. Mutations introduced in the “6D” are colored as blue; those in the “7D” and “8D” as red. Tan box refers to transmembrane domain (TMD). **b,** Representative current traces of AtSLAC1 wild-type (WT, black), 6D (blue), WT-GFP (green fluorescent protein, green) and 6D-GFP (cyan) evoked by voltage stimulations of -100 mV to +100 mV with 20 mV increments. **c,** I-V relationships of the indicated channels. Each data was represented as mean ± standard error of the mean. **d,** Current densities of the indicated channels in **c** at +60 mV. Number of observations are indicated in parentheses. Independent *t*-test was used for comparisons and the P values are indicated. **e-f,** I-V relationships of the indicated channels. Each data was represented as mean ± standard error of the mean. **g,** Current densities of indicated channels in **e-f** at +60 mV. Number of observations are indicated in parentheses. One-way ANOVA was used for comparisons. Different letters indicated significant difference ($P < 0.05$) from others by Dunnett’s T3 multiple comparisons test.

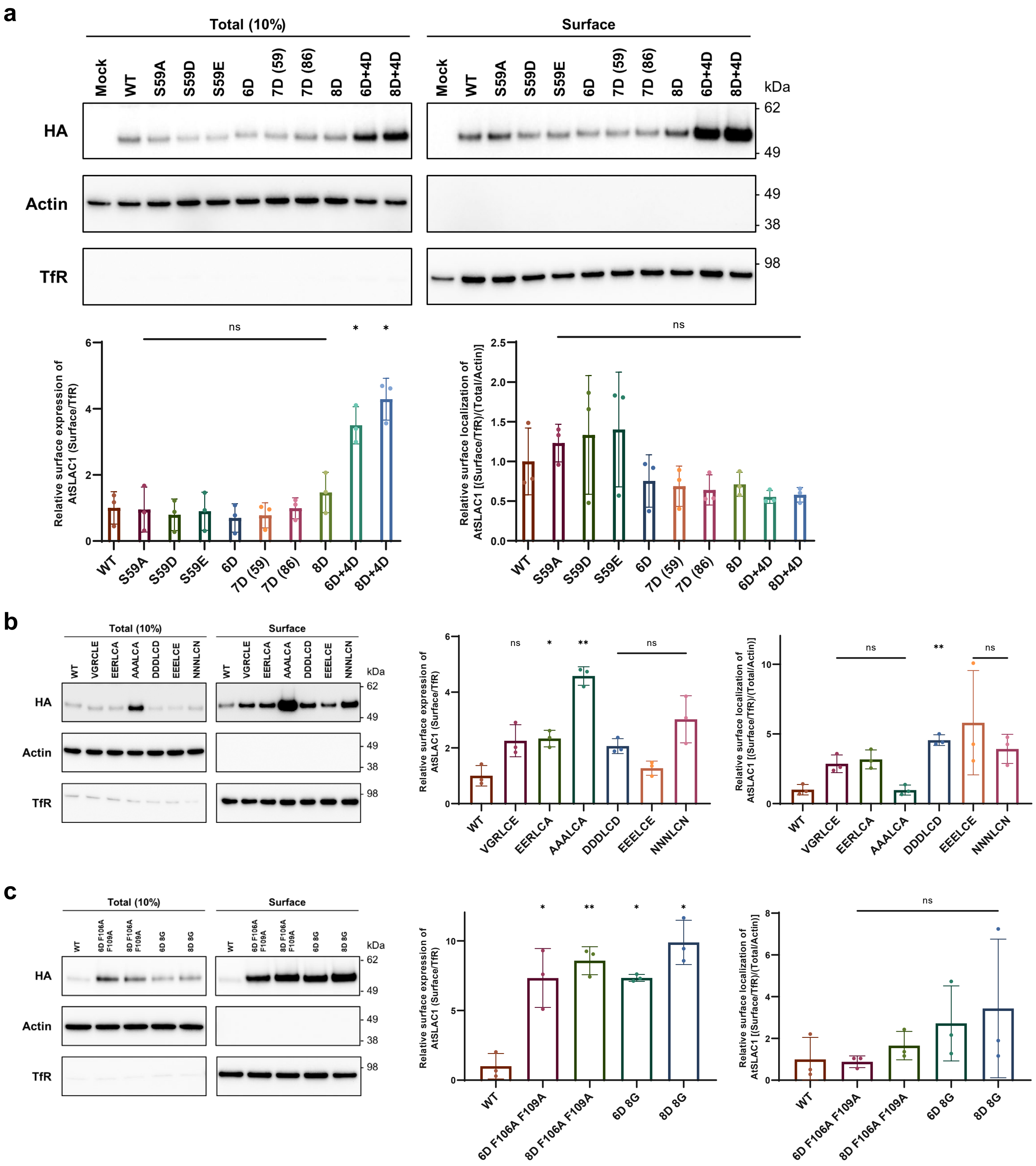

Supplementary Figure 2. Expression levels of AtSLAC1 WT and its mutants.

Surface expression and localization of AtSLAC1 WT and its mutants were measured by immunoblot analysis of biotinylated AtSLAC1 on cell surface. Each data from triplicated samples was represented as mean ± standard deviation. One-way ANOVA was used for comparisons against AtSLAC1 WT by Dunnett's T3 multiple comparisons test. P values of **a** are > 0.9999, 0.9985, > 0.9999, 0.9770, 0.9947, > 0.9999, 0.9232, 0.0246, 0.0116, 0.9690, 0.9896, 0.9676, 0.9783, 0.8934, 0.7923, 0.8873, 0.6146 and 0.6570, respectively. P values of **b** are 0.1725, 0.0340, 0.0010, 0.0629, 0.8502, 0.1135, 0.0815, 0.0612, > 0.9999, 0.0015, 0.4231 and 0.0719, respectively. P values of **c** are 0.0500, 0.0021, 0.0180, 0.0103, 0.9991, 0.8293, 0.5709 and 0.6923, respectively. Ns, not statistically significant; *, P value ≤ 0.05; **, P value ≤ 0.01.

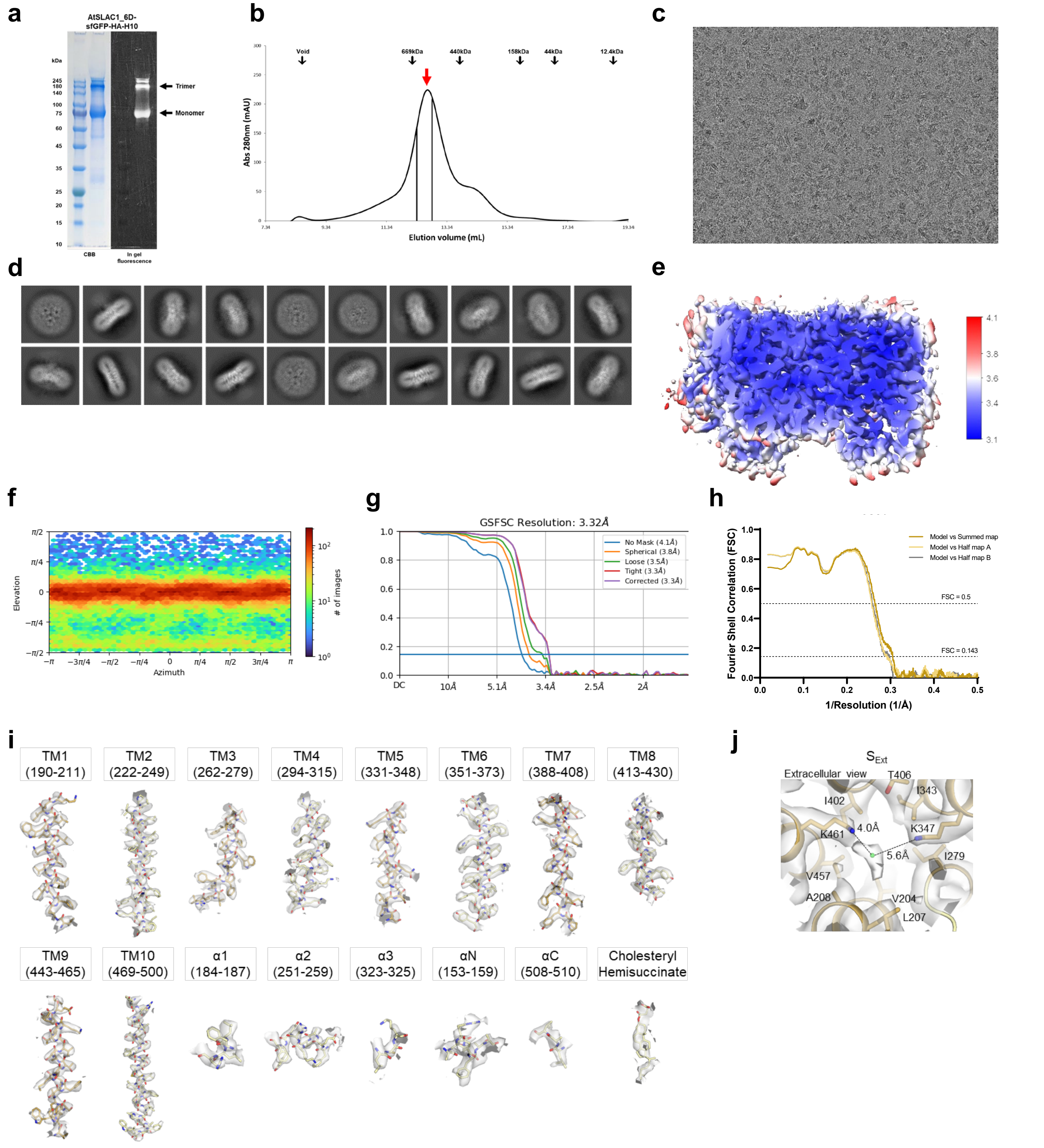

Supplementary Figure 3. Cryo-EM analysis of the closed structure of AtSLAC1 6D.

a, A representative SDS-PAGE gel stained by Coomassie blue (*left*) and detected by fluorescence (*right*) to show the purity of AtSLAC1 6D-sfGFP-HA-H_{10} fusion protein. **b,** A representative size exclusion chromatogram of AtSLAC1 6D-sfGFP-HA-H_{10}. A peak containing the fusion protein is indicated by a red arrow. Molecular mass markers are shown with their molecular masses in kDa. **c,** A representative micrograph of AtSLAC1 6D-sfGFP-HA-H_{10}. **d,** A representative 2D classified images. **e,** Local resolution map. **f,** Euler angle distribution. **g,** Gold-standard Fourier shell correlation (GSFSC) curves for overall map. Overall resolution reaches 3.32 Å by a threshold value of 0.143 indicated by a blue horizontal line. **h,** Map-to-model Fourier shell correlative (FSC) curve. **i,** Cryo-EM map densities of secondary structural elements and cholesteryl hemisuccinate. **j,** Density of a putative chloride ion site in the extracellular side of the ion conductance pore (S_{Ext}) is modeled as a chloride ion and depicted as a green sphere. Carbon atoms are colored as yellow, oxygen as red, nitrogen as blue and sulfur as yellow.

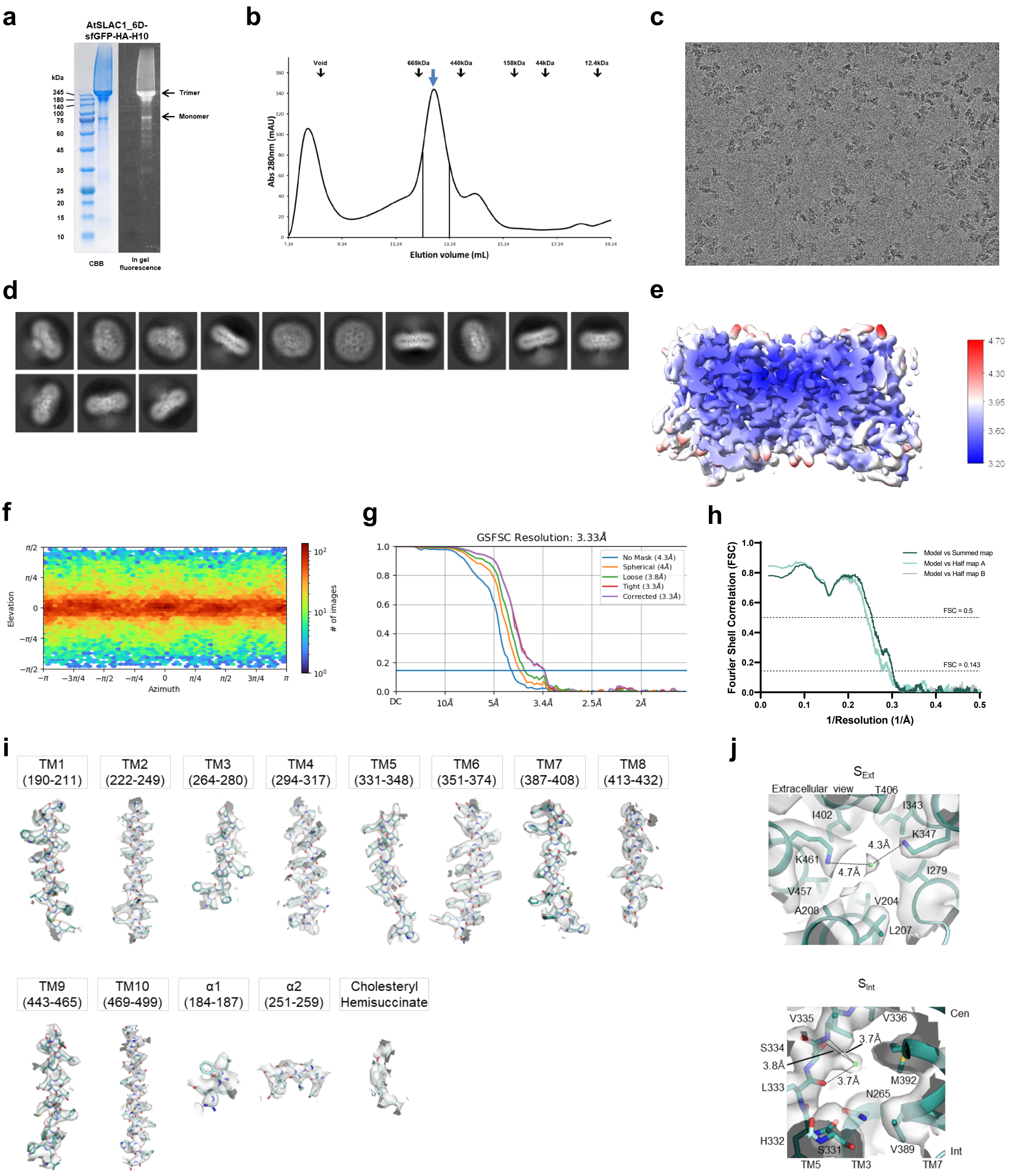

Supplementary Figure 4. Cryo-EM analysis of the open structure of AtSLAC1 6D.

a, A representative SDS-PAGE gel stained by Coomassie blue (*left*) and detected by fluorescence (*right*) to show the purity of AtSLAC1 6D-sfGFP-HA-H_{10} fusion protein. **b,** A representative size exclusion chromatogram of AtSLAC1 6D-sfGFP-HA-H_{10}. A peak containing the fusion protein is indicated by a blue arrow. Molecular mass markers are shown with their molecular masses in kDa. **c,** A representative micrograph of AtSLAC1 6D-sfGFP-HA-H_{10}. **d,** A representative 2D classified images. **e,** Local resolution map. **f,** Euler angle distribution. **g,** Overall resolution reaches 3.33 Å by a threshold value of 0.143 indicated by a blue horizontal line. **h,** Map-to-model Fourier shell correlative (FSC) curve. **i,** Cryo-EM map densities of secondary structural elements and cholesteryl hemisuccinate. **j,** Density of a putative chloride ion site in the intracellular side (S_{Int}) and the other in the extracellular side of the ion conductance pore (S_{Ext}) are modeled as chloride ions and depicted as green spheres. Chloride ions are depicted as green spheres. Carbon atoms are colored as teal, oxygen as red, nitrogen as blue and sulfur as yellow.

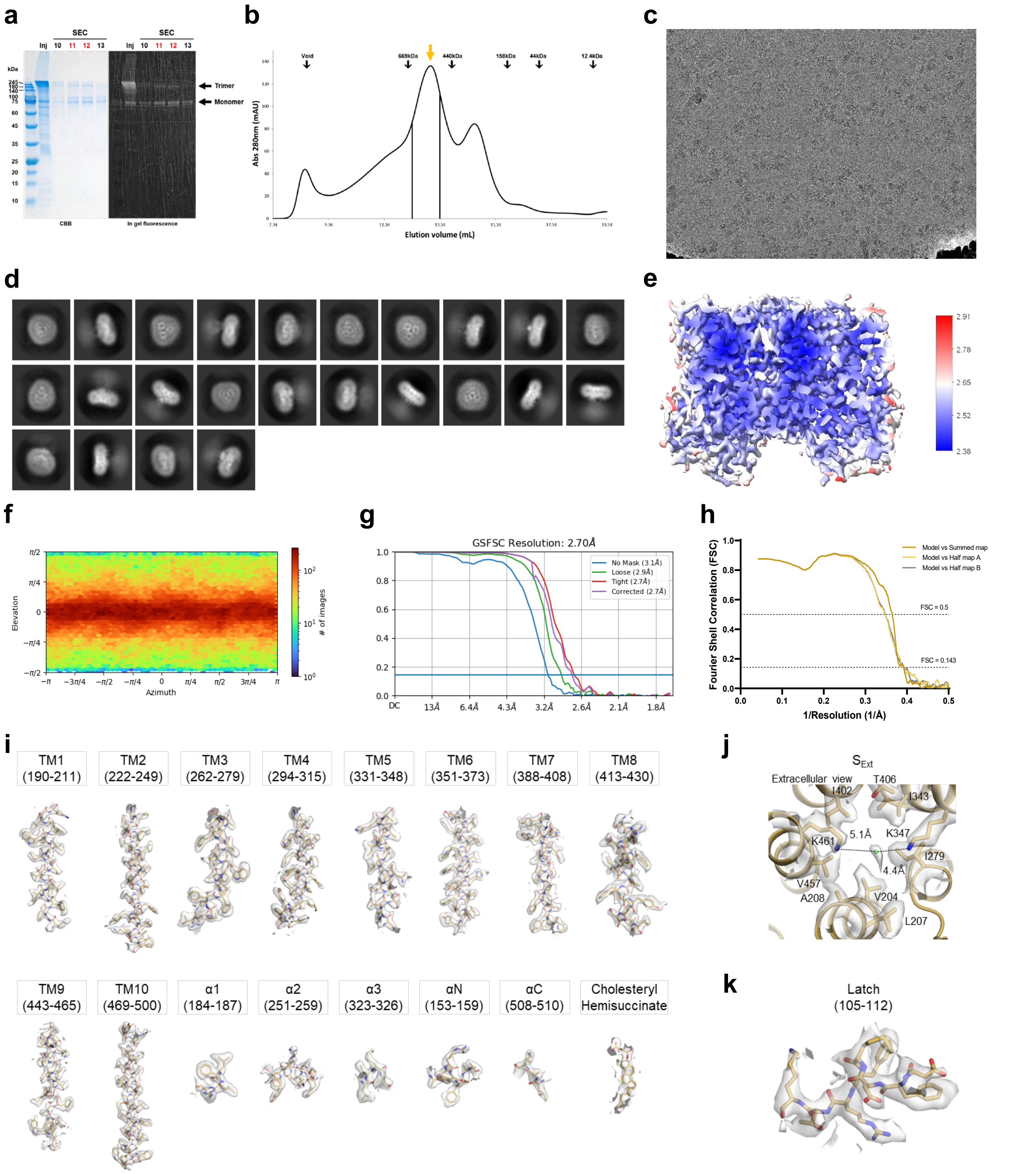

Supplementary Figure 5. Cryo-EM analysis of the closed structure of AtSLAC1 8D.

a, A representative SDS-PAGE gel stained by Coomassie blue (*left*) and detected by fluorescence (*right*) to show the size exclusion chromatography (SEC) and purity of AtSLAC1 8D-sfGFP-HA-H_{10} fusion protein. **b,** A representative size exclusion chromatogram of AtSLAC1 8D-sfGFP-HA-H_{10}. A peak containing the fusion protein is indicated by a red arrow. Molecular mass markers are shown with their molecular masses in kDa. **c,** A representative micrograph of AtSLAC1 8D-sfGFP-HA-H_{10}. **d,** A representative 2D classified images. **e,** Local resolution map. **f,** Euler angle distribution. **g,** Gold-standard Fourier shell correlation (GSFSC) curves for overall map. Overall resolution reaches 2.70 Å by a threshold value of 0.143 indicated by a blue horizontal line. **h,** Map-to-model Fourier shell correlative (FSC) curve. **i,** Cryo-EM map densities of secondary structural elements and cholesteryl hemisuccinate. **j,** Density of putative chloride ion site in the extracellular side of the ion conductance pore ($Site_{Ext}$) is modeled as chloride ion and depicted as green sphere. **k,** Cryo-EM density of latch are shown as gray surface, overlaid with the final refined structural model in sticks. Carbon atoms are colored as yellow, oxygen as red, nitrogen as blue and sulfur as yellow.

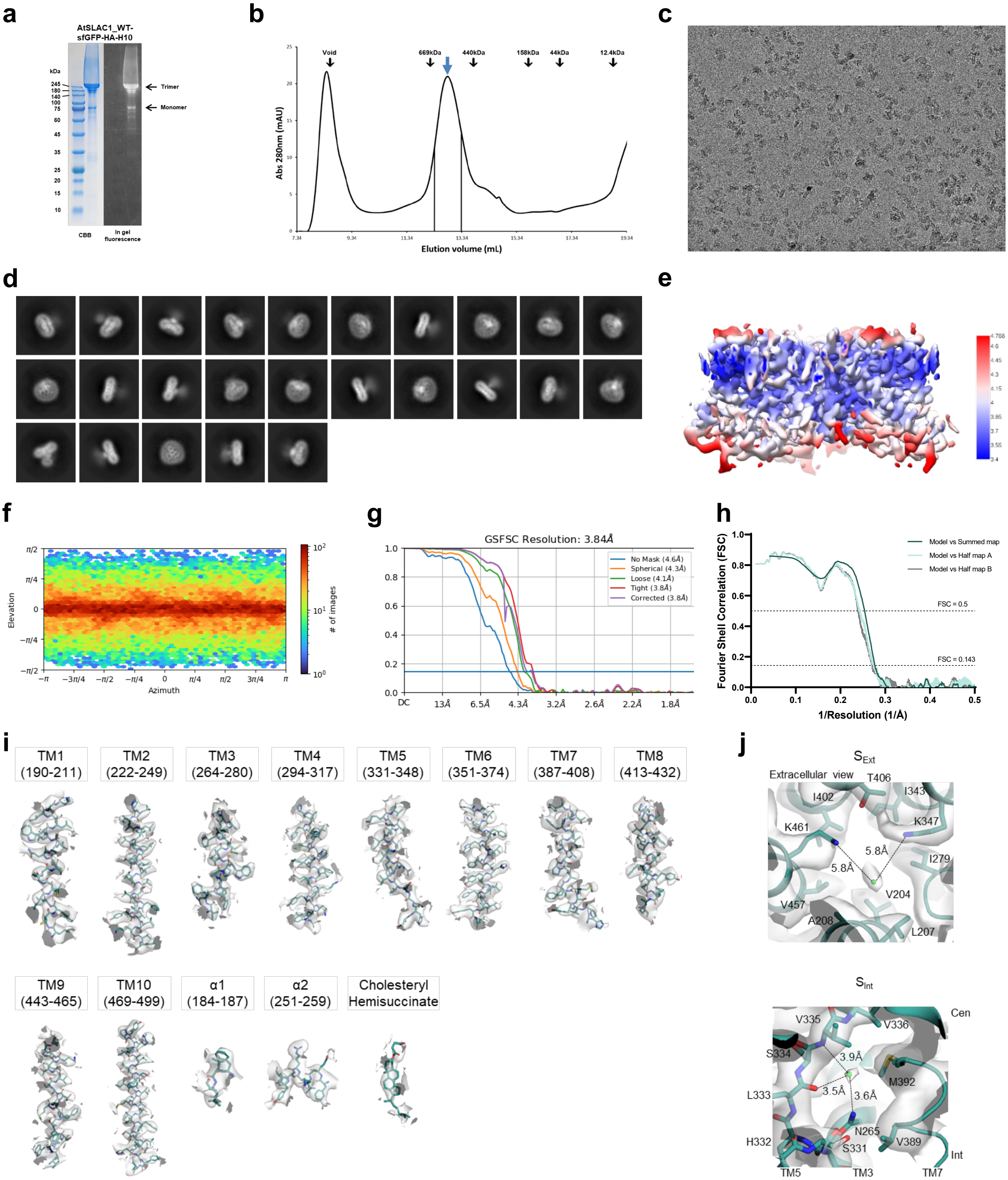

Supplementary Figure 6. Cryo-EM analysis of the open structure of AtSLAC1.

a, A representative SDS-PAGE gel stained by Coomassie blue (*left*) and detected by fluorescence (*right*) to show the size exclusion chromatography (SEC) and purity of AtSLAC1-sfGFP-HA-H_{10} fusion protein. **b,** A representative size exclusion chromatogram of AtSLAC1-sfGFP-HA-H_{10}. A peak containing the fusion protein is indicated by a red arrow. Molecular mass markers are shown with their molecular masses in kDa. **c,** A representative micrograph of AtSLAC1-sfGFP-HA-H_{10}. **d,** A representative 2D classified images. **e,** Local resolution map. **f,** Euler angle distribution. **g,** Gold-standard Fourier shell correlation (GSFSC) curves for overall map. Overall resolution reaches 3.84 Å by a threshold value of 0.143 indicated by a blue horizontal line. **h,** Map-to-model Fourier shell correlative curve. **i,** Cryo-EM map densities of secondary structural elements and cholesteryl hemisuccinate. **j,** Density of a putative chloride ion site in the intracellular side (S_{Int}) and the other in the extracellular side of the ion conductance pore (S_{Ext}) are modeled as chloride ions and depicted as green spheres. Chloride ions are depicted as green spheres. Carbon atoms are colored as teal, oxygen as red, nitrogen as blue and sulfur as yellow.

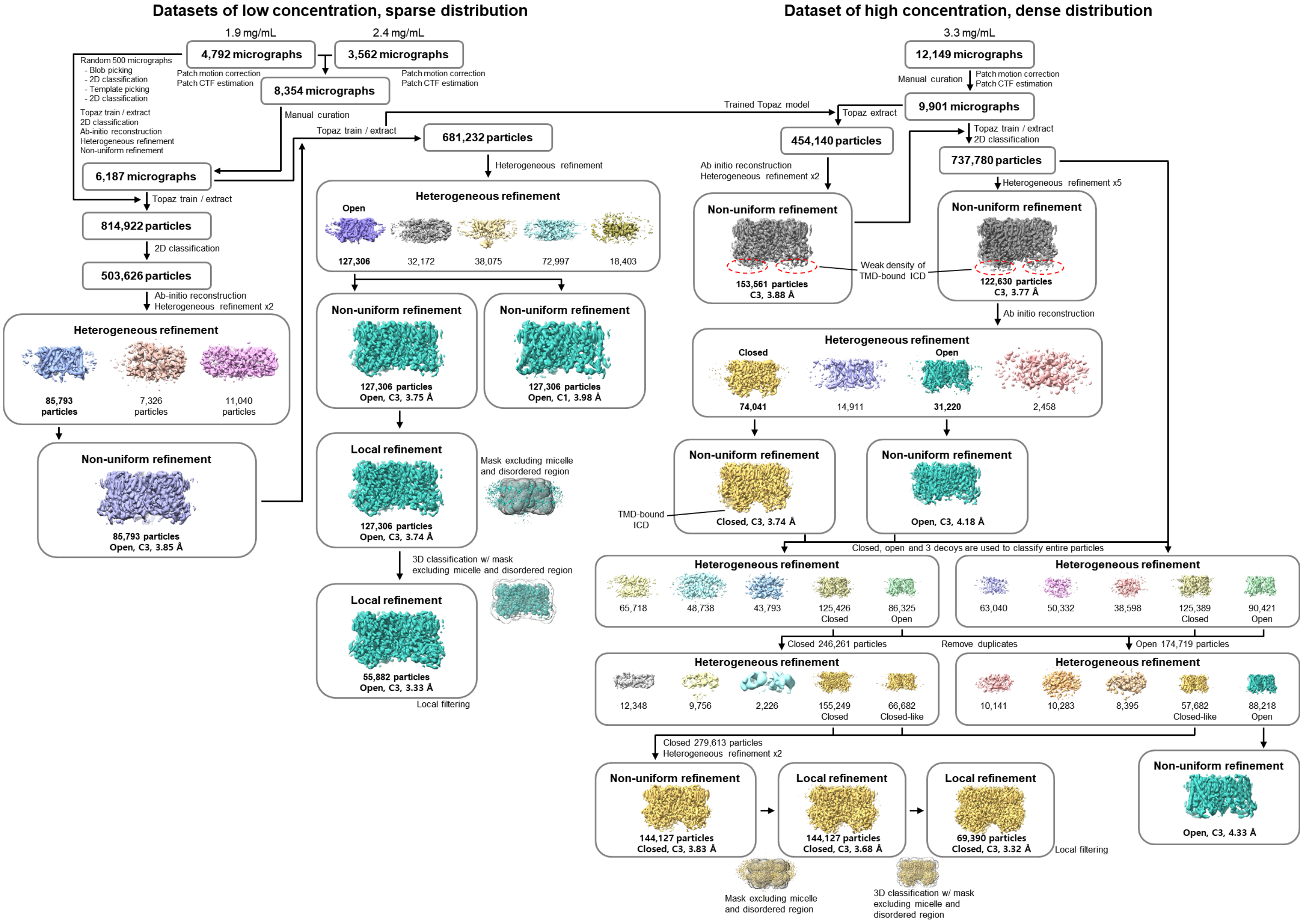

Supplementary Figure 7. Cryo-EM data processing workflow of the open and closed structures of AtSLAC1 6D.

Cryo-EM data processing scheme of AtSLAC1 6D-sfGFP-HA-H_{10} from different datasets using cryoSPARC. See Methods for details.

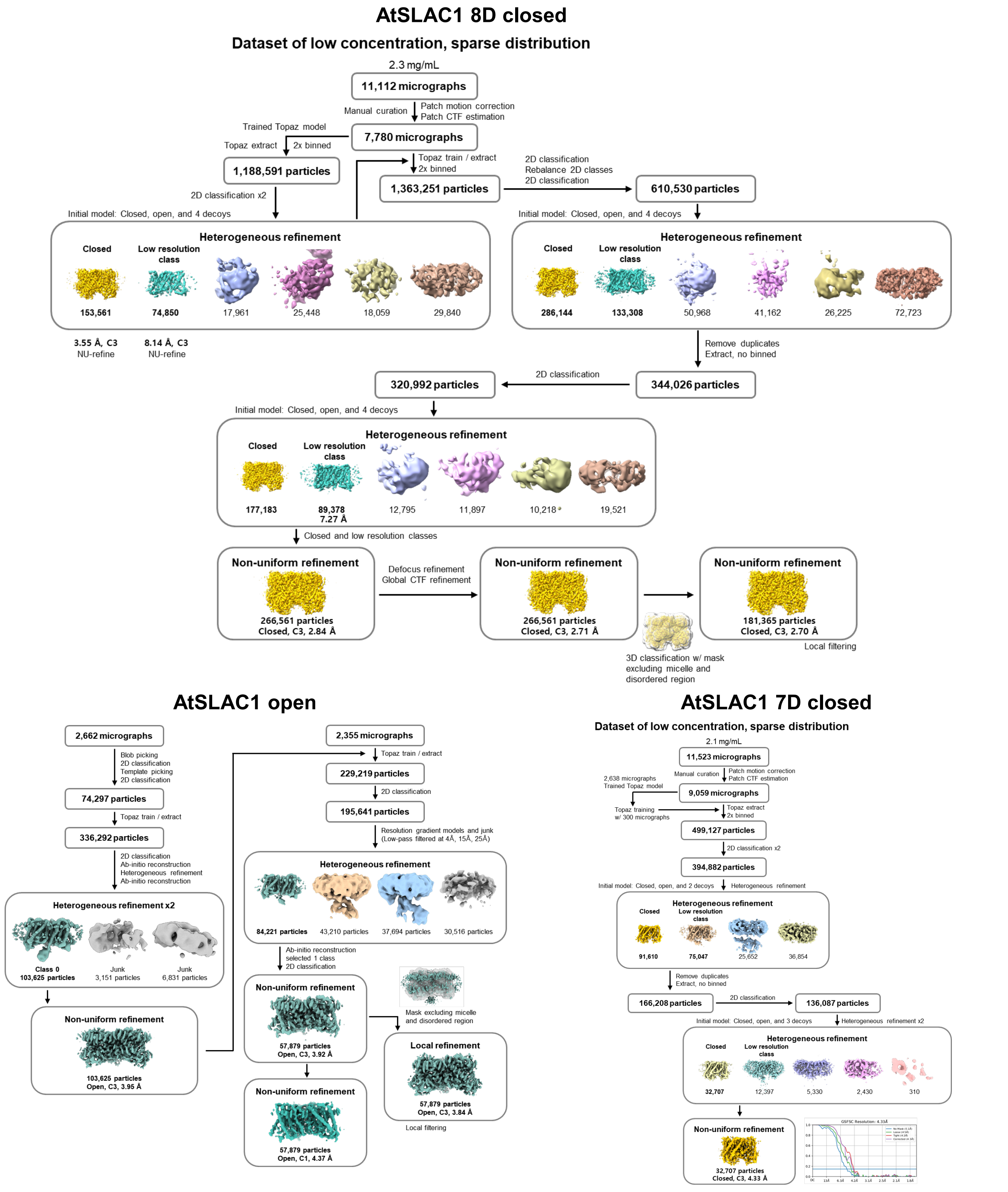

Supplementary Figure 8. Cryo-EM data processing workflows of the open structure of AtSLAC1 and the closed structures of AtSLAC1 7D and 8D.

Cryo-EM data processing scheme of AtSLAC1-sfGFP-HA-H_{10}, AtSLAC1 7D-sfGFP-HA-H_{10} and AtSLAC1 8D-sfGFP-HA-H_{10} using cryoSPARC. See Methods for details.

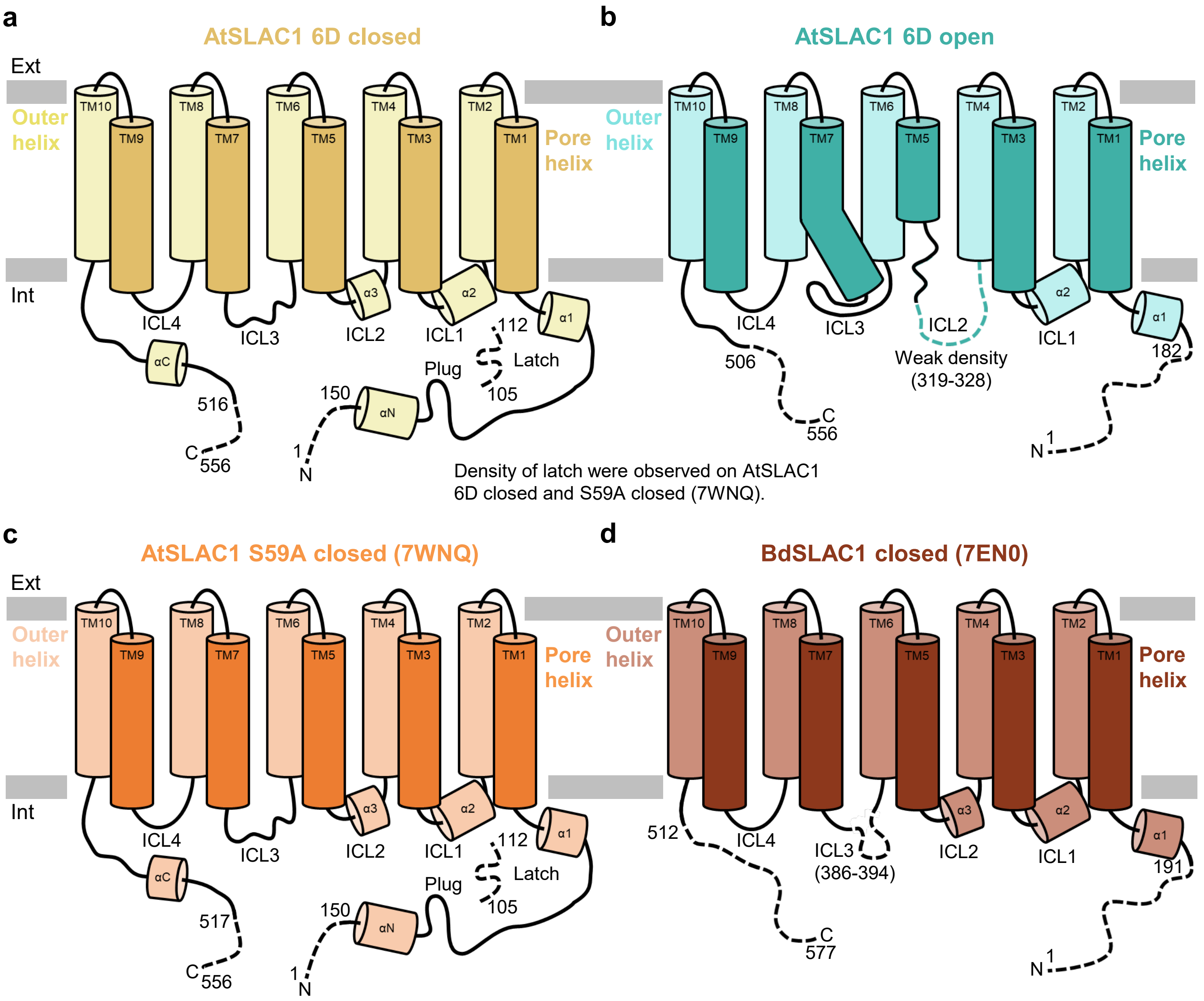

Supplementary Figure 9. Topologies of the open and closed SLAC1 structures.

a-d, Topologies of the open (teal) and closed (yellow) structures of AtSLAC1 6D, that of the closed AtSLAC1 S59A structure (Orange, PDB ID: 7WNQ) and that of the closed BdSLAC1 structure (Brown, PDB ID: 7EN0). Dashed lines indicate flexible and unmodeled regions.

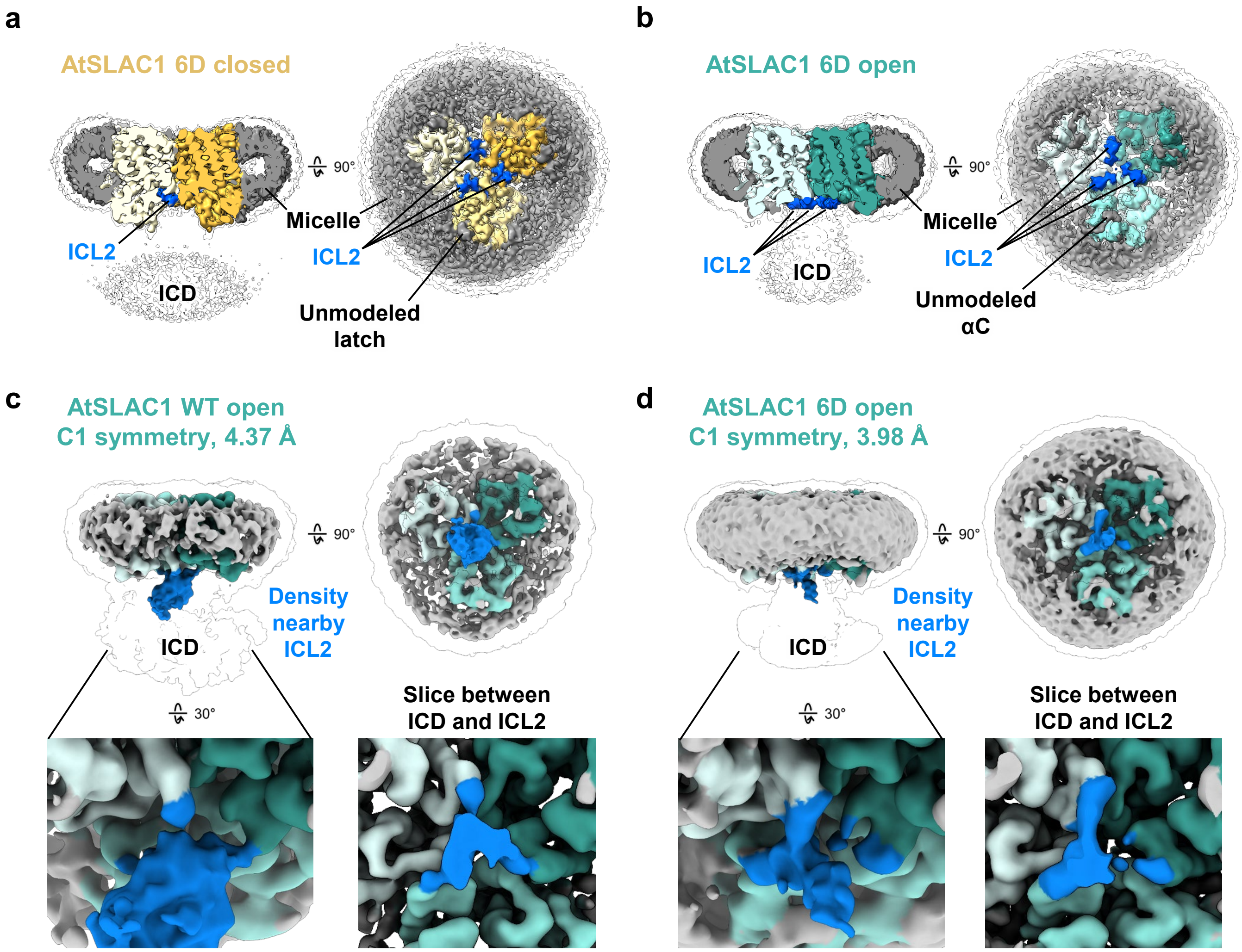

Supplementary Figure 10. Interactions between ICL2 and ICD.

a-b, Cross-sectional side and intracellular view of cryo-EM maps for AtSLAC1 6D closed and open structures. ICLD, intracellular domain; ICL2, intracellular loop 2. **c-d,** Cryo-EM maps of AtSLAC1 WT and 6D open structures without applied symmetry. Unsharpened maps are used to visualize ordered regions, transmembrane domain (TMD), plug, micelle (gray) and densities around ICL2 (blue). One protomer with TMD and plug densities of modeled regions is colored as yellow for the closed structure and teal for the open structure, respectively. Densities of other protomers are colored as light colors. Unsharpened maps at a lower contour level (black border lines) show densities of micelle and disordered intracellular regions.

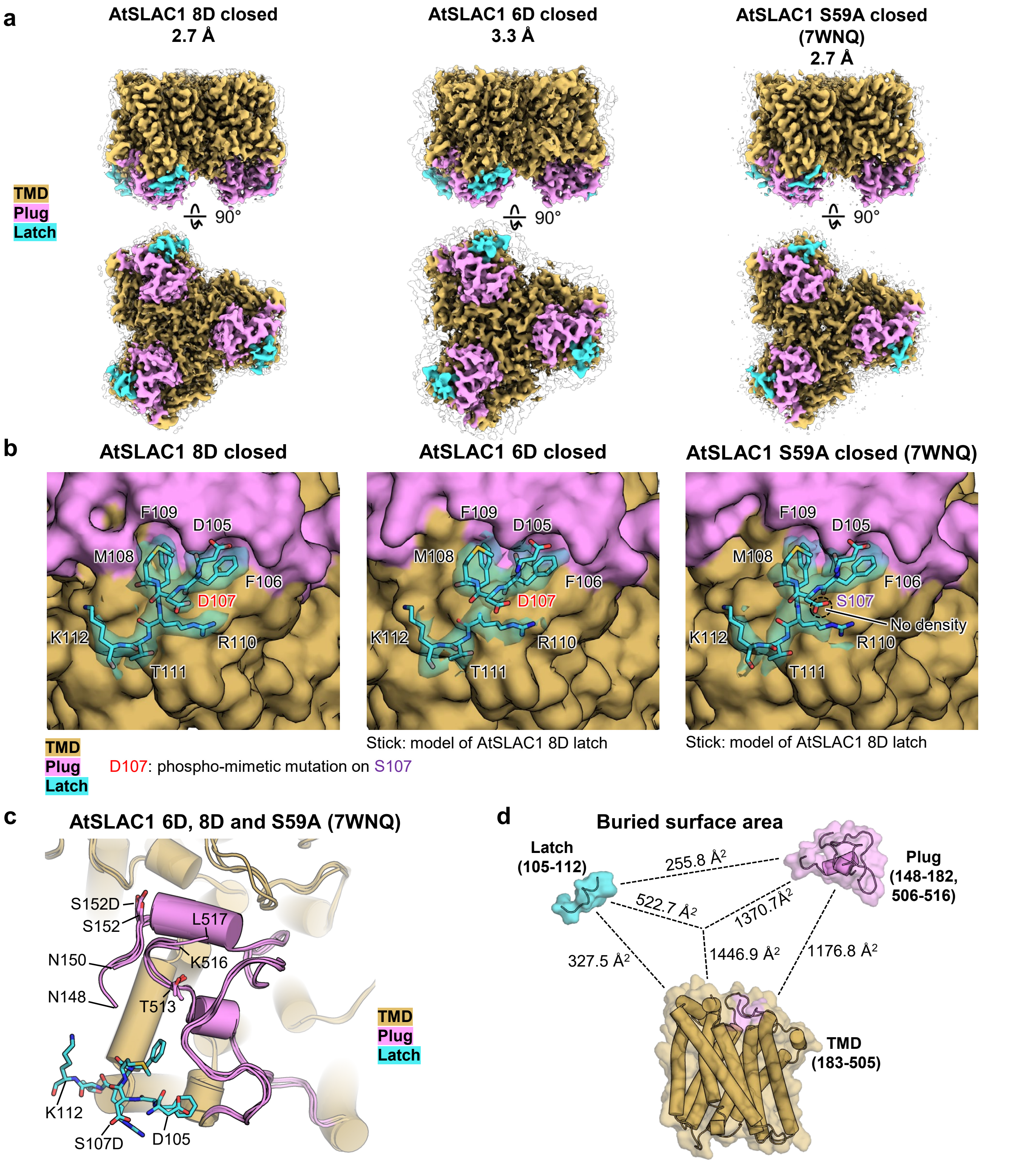

Supplementary Figure 11. Previously unidentified density (latch) can be found on AtSLAC1 closed structures.

a, Side and intracellular views of closed state maps: AtSLAC1 6D, 8D and S59A (PDB: 7WNQ). Transmembrane domain (TMD), plug and latch are colored with yellow, pink and cyan, respectively. **b,** The latch density in each closed state are shown as transparent surface. The latch model of AtSLAC1 8D is used to represent the latch of other closed structures. TMD and plug are shown as surface of model. **c,** Comparison of latch and plug among three closed structures. N-, C-terminal residues and two phosphomimetic mutations (S107D and S152D) are labeled. **d,** Buried surface area among latch, plug and TMD. Buried surface areas on each interface are calculated by PISA[1].

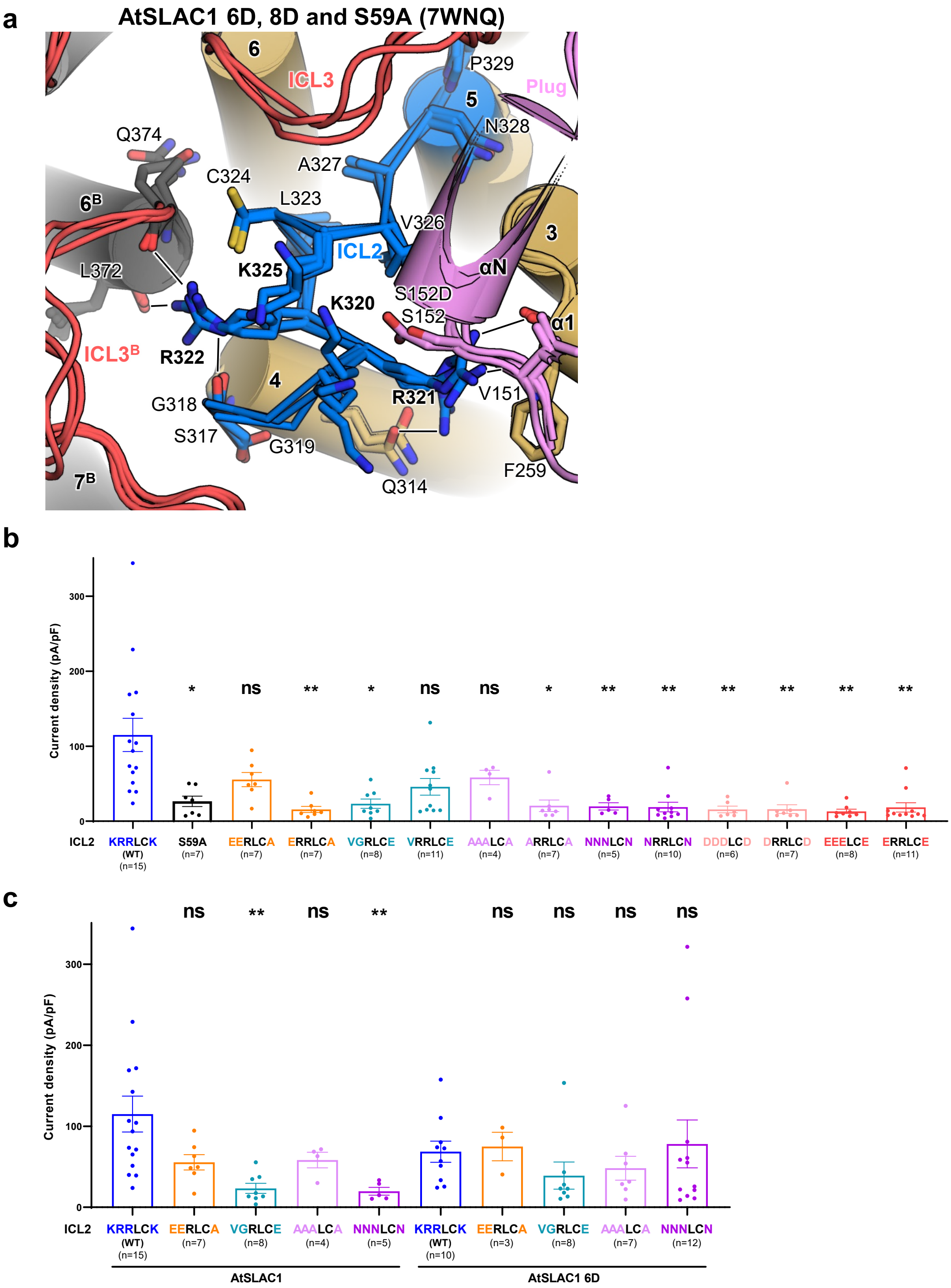

Supplementary Figure 12. Closed conformation of ICL2 and activation of SLAC1.

a, ICL2 in closed conformation of AtSLAC1 6D, 8D and S59A (PDB: 7WNQ). All structures are depicted as cartoon representations. Numbers indicate TM numbers. ICL2 is shown as stick and ribbon, and residues neighboring with arginine residues of ICL2 are shown as stick. Interaction between arginine and neighboring residues are indicated by lines. **b-c,** Current densities of indicated channels at +60 mV. Each data was represented as mean ± standard error of the mean. Number of observations are indicated in the parenthesis. One-way ANOVA was used for comparisons against AtSLAC1 WT or 6D by Dunnett's T3 multiple comparisons test. P values of **b** are 0.0168, 0.2349, 0.0061, 0.0127, 0.1237, 0.2953, 0.0106, 0.0092, 0.0087, 0.0063, 0.0065, 0.0053 and 0.0082, respectively. P values of **c** are 0.1600, 0.0081, 0.2050, 0.0059, > 0.9999, 0.7534, 0.9280 and > 0.9999, respectively. Ns, not statistically significant; *, P value ≤ 0.05; **, P value ≤ 0.01.

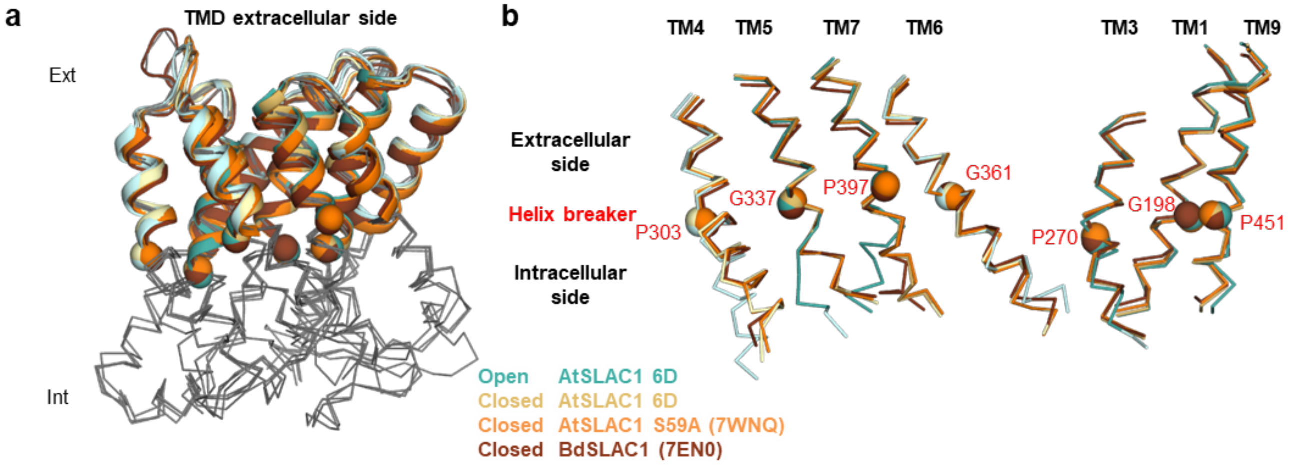

c

Cα RMSD (Å): 6- (red); 4-6 (orange); 2-4 (yellow); 1-2 (green); 0-1 (blue)

☐ : Flexible region (outlined in red)

Whole TMD

TMD w/o unmodelled regions (182-318, 329-375, 385-500)

Cα RMSD (Å)	BdSLAC1 closed (7EN0)	AtSLAC1 S59A closed (7WNQ)	AtSLAC1 6D closed	AtSLAC1 6D open
BdSLAC1 closed (7EN0)		0.8	0.9	1.1
AtSLAC1 S59A closed (7WNQ)			0.4	0.7
AtSLAC1 6D closed				0.6
AtSLAC1 6D open				

TMD extracellular side

TMD extracellular side (198-235, 270-303, 337-361, 397-421, 451-484)

Cα RMSD (Å)	BdSLAC1 closed (7EN0)	AtSLAC1 S59A closed (7WNQ)	AtSLAC1 6D closed	AtSLAC1 6D open
BdSLAC1 closed (7EN0)		1.2	1.2	1.4
AtSLAC1 S59A closed (7WNQ)			0.5	0.9
AtSLAC1 6D closed				0.7
AtSLAC1 6D open				

TMs extracellular side (198-211, 221-235, 270-280, 294-303, 337-348, 351-361, 397-408, 413-421, 451-465, 469-484)

Cα RMSD (Å)	BdSLAC1 closed (7EN0)	AtSLAC1 S59A closed (7WNQ)	AtSLAC1 6D closed	AtSLAC1 6D open
BdSLAC1 closed (7EN0)		0.8	0.8	1.0
AtSLAC1 S59A closed (7WNQ)			0.4	0.7
AtSLAC1 6D closed				0.6
AtSLAC1 6D open				

ECLs (212-220, 281-293, 349-350, 409-412, 466-468)

Cα RMSD (Å)	BdSLAC1 closed (7EN0)	AtSLAC1 S59A closed (7WNQ)	AtSLAC1 6D closed	AtSLAC1 6D open
BdSLAC1 closed (7EN0)		2.3	2.2	2.4
AtSLAC1 S59A closed (7WNQ)			0.7	1.2
AtSLAC1 6D closed				1.1
AtSLAC1 6D open				

TMD intracellular side

Pore helix

TM1 intracellular side (190-197)

Cα RMSD (Å)	BdSLAC1 closed (7EN0)	AtSLAC1 S59A closed (7WNQ)	AtSLAC1 6D closed	AtSLAC1 6D open
BdSLAC1 closed (7EN0)		0.7	1.0	1.2
AtSLAC1 S59A closed (7WNQ)			0.5	0.8
AtSLAC1 6D closed				0.5
AtSLAC1 6D op				

TM3 intracellular side (262-269)

Cα RMSD (Å)	BdSLAC1 closed (7EN0)	AtSLAC1 S59A closed (7WNQ)	AtSLAC1 6D closed	AtSLAC1 6D open
BdSLAC1 closed (7EN0)		0.6	0.4	1.1
AtSLAC1 S59A closed (7WNQ)			0.6	1.1
AtSLAC1 6D closed				1.0
AtSLAC1 6D open				

TM5 intracellular side (329-336)

Cα RMSD (Å)	BdSLAC1 closed (7EN0)	AtSLAC1 S59A closed (7WNQ)	AtSLAC1 6D closed	AtSLAC1 6D open
BdSLAC1 closed (7EN0)		0.4	0.5	6.5
AtSLAC1 S59A closed (7WNQ)			0.5	6.4
AtSLAC1 6D closed				6.5
AtSLAC1 6D open				

TM7 intracellular side (387-396)

Cα RMSD (Å)	BdSLAC1 closed (7EN0)	AtSLAC1 S59A closed (7WNQ)	AtSLAC1 6D closed	AtSLAC1 6D open
BdSLAC1 closed (7EN0)		2.0	1.8	4.2
AtSLAC1 S59A closed (7WNQ)			0.4	5.3
AtSLAC1 6D closed				5.1
AtSLAC1 6D open				

TM9 intracellular side (443-450)

Cα RMSD (Å)	BdSLAC1 closed (7EN0)	AtSLAC1 S59A closed (7WNQ)	AtSLAC1 6D closed	AtSLAC1 6D open
BdSLAC1 closed (7EN0)		1.0	1.0	1.3
AtSLAC1 S59A closed (7WNQ)			0.6	1.0
AtSLAC1 6D closed				1.0
AtSLAC1 6D open				

ICL

ICL1 (251-261)

Cα RMSD (Å)	BdSLAC1 closed (7EN0)	AtSLAC1 S59A closed (7WNQ)	AtSLAC1 6D closed	AtSLAC1 6D open
BdSLAC1 closed (7EN0)		0.7	0.9	1.4
AtSLAC1 S59A closed (7WNQ)			0.5	1.1
AtSLAC1 6D closed				0.8
AtSLAC1 6D open				

ICL2 (319-328)

Cα RMSD (Å)	BdSLAC1 closed (7EN0)	AtSLAC1 S59A closed (7WNQ)	AtSLAC1 6D closed	AtSLAC1 6D open
BdSLAC1 closed (7EN0)		1.0	1.2	n.d.
AtSLAC1 S59A closed (7WNQ)			0.7	n.d.
AtSLAC1 6D closed				n.d.
AtSLAC1 6D open				

ICL3 (375-386)

Cα RMSD (Å)	BdSLAC1 closed (7EN0)	AtSLAC1 S59A closed (7WNQ)	AtSLAC1 6D closed	AtSLAC1 6D open
BdSLAC1 closed (7EN0)		n.d.	n.d.	n.d.
AtSLAC1 S59A closed (7WNQ)			1.0	11.4
AtSLAC1 6D closed				11.7
AtSLAC1 6D open				

ICL4 (433-442)

Cα RMSD (Å)	BdSLAC1 closed (7EN0)	AtSLAC1 S59A closed (7WNQ)	AtSLAC1 6D closed	AtSLAC1 6D open
BdSLAC1 closed (7EN0)		3.6	3.7	4.6
AtSLAC1 S59A closed (7WNQ)			0.5	4.8
AtSLAC1 6D closed				4.7
AtSLAC1 6D open				

Outer helix

TM10 intracellular side (485-500)

Cα RMSD (Å)	BdSLAC1 closed (7EN0)	AtSLAC1 S59A closed (7WNQ)	AtSLAC1 6D closed	AtSLAC1 6D open
BdSLAC1 closed (7EN0)		2.0	2.1	2.2
AtSLAC1 S59A closed (7WNQ)			0.4	1.1
AtSLAC1 6D closed				1.0
AtSLAC1 6D open				

TM2 intracellular side (236-250)

Cα RMSD (Å)	BdSLAC1 closed (7EN0)	AtSLAC1 S59A closed (7WNQ)	AtSLAC1 6D closed	AtSLAC1 6D open
BdSLAC1 closed (7EN0)		1.1	1.2	1.2
AtSLAC1 S59A closed (7WNQ)			0.4	0.6
AtSLAC1 6D closed				0.4
AtSLAC1 6D open				

TM4 intracellular side (304-318)

Cα RMSD (Å)	BdSLAC1 closed (7EN0)	AtSLAC1 S59A closed (7WNQ)	AtSLAC1 6D closed	AtSLAC1 6D open
BdSLAC1 closed (7EN0)		1.5	1.5	3.3
AtSLAC1 S59A closed (7WNQ)			0.6	4.0
AtSLAC1 6D closed				3.9
AtSLAC1 6D open				

TM6 intracellular side (362-374)

Cα RMSD (Å)	BdSLAC1 closed (7EN0)	AtSLAC1 S59A closed (7WNQ)	AtSLAC1 6D closed	AtSLAC1 6D open
BdSLAC1 closed (7EN0)		0.8	0.8	1.6
AtSLAC1 S59A closed (7WNQ)			0.4	2.0
AtSLAC1 6D closed				2.0
AtSLAC1 6D open				

TM8 intracellular side (422-432)

Cα RMSD (Å)	BdSLAC1 closed (7EN0)	AtSLAC1 S59A closed (7WNQ)	AtSLAC1 6D closed	AtSLAC1 6D open
BdSLAC1 closed (7EN0)		2.1	2.1	1.0
AtSLAC1 S59A closed (7WNQ)			0.2	2.4
AtSLAC1 6D closed				2.3
AtSLAC1 6D open				

Supplementary Figure 13. Conformational changes on intracellular side and Cα r.m.s.d. values of SLAC1 structures.

a, Superposition of SLAC1 structures. Extracellular side regions are depicted as cartoon with the corresponding color of each structure. Other regions are depicted as gray ribbons. **b,** Superposition of TMs that contain helix breakers. TMs of the open and closed structures are depicted as ribbon representations. TMs can be divided into intracellular and extracellular side by the helix breakers. Positions of the helix breakers are indicated by Cα spheres and labeled as red letters. **c,** Tables showing representative Cα r.m.s.d. (Å) between specified region of two structures. R.m.s.d. values smaller than 1.0 Å are colored as blue, those smaller than 2.0 Å as green, those smaller than 4.0 Å as yellow, smaller than 6.0 Å as orange and the remainders as red. All r.m.s.d. values are rounded off to the first decimal place.

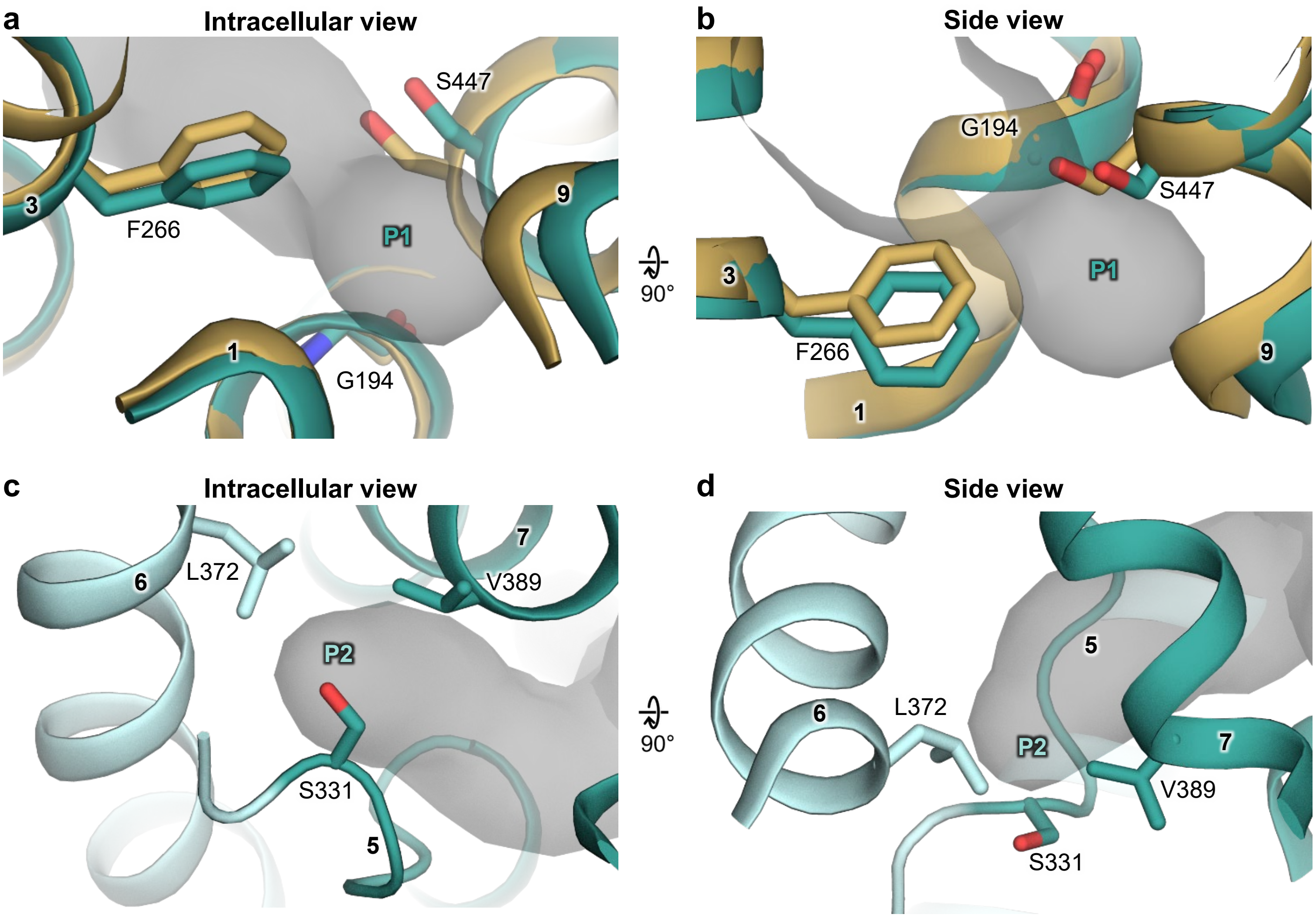

Supplementary Figure 14. Entries of pores P1 and P2 in closed and structures.

a-b, Intracellular and side view of P1 entry in closed and open structures. The closed structure is colored as yellow and the open structure as teal (pore helices) and cyan (the rest). Three pore-lining residues at constriction are depicted as stick models. Pore volume of the open structure is shown as gray surface. Numbers indicate TM numbers. **c-d,** Intracellular and side view of P2 entry in open states. Three pore-lining residues at constriction are depicted as stick models. Pore volume of open state is shown as gray surface.

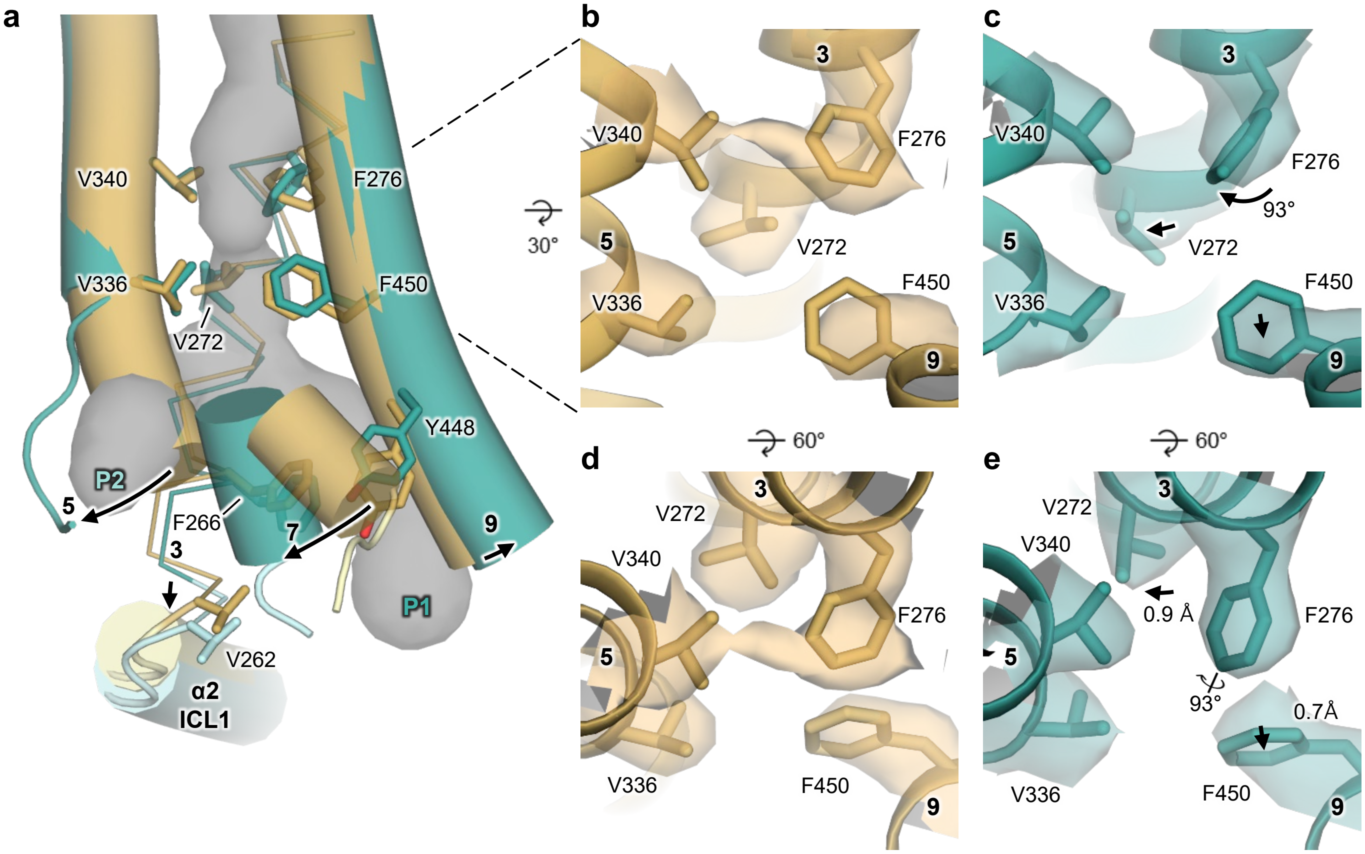

Supplementary Figure 15. Central gate in closed and open structures.

a. Conformational changes that related to central gate opening during closed to open transition of AtSLAC1 6D. The closed structure is colored as yellow (pore helices) and light yellow (the rest) and the open structure as teal (pore helices) and cyan (the rest). Pore volume of the open structure is shown as gray surface. Numbers indicate TM numbers. Movements and rotations of the AtSLAC1 6D open structure in reference to those of the closed structure are indicated with black thick arrows and angles. **b-e,** Comparison of the central gate in the closed (**b, d**) and open (**c, e**) structures of AtSLAC1 6D. Five central gate residues (V272, F276, V336, V340, and F450) are shown as stick and corresponding map density. The panel **d** is a 60-degree rotated view of **b** and the panel **e** is that of **c**.

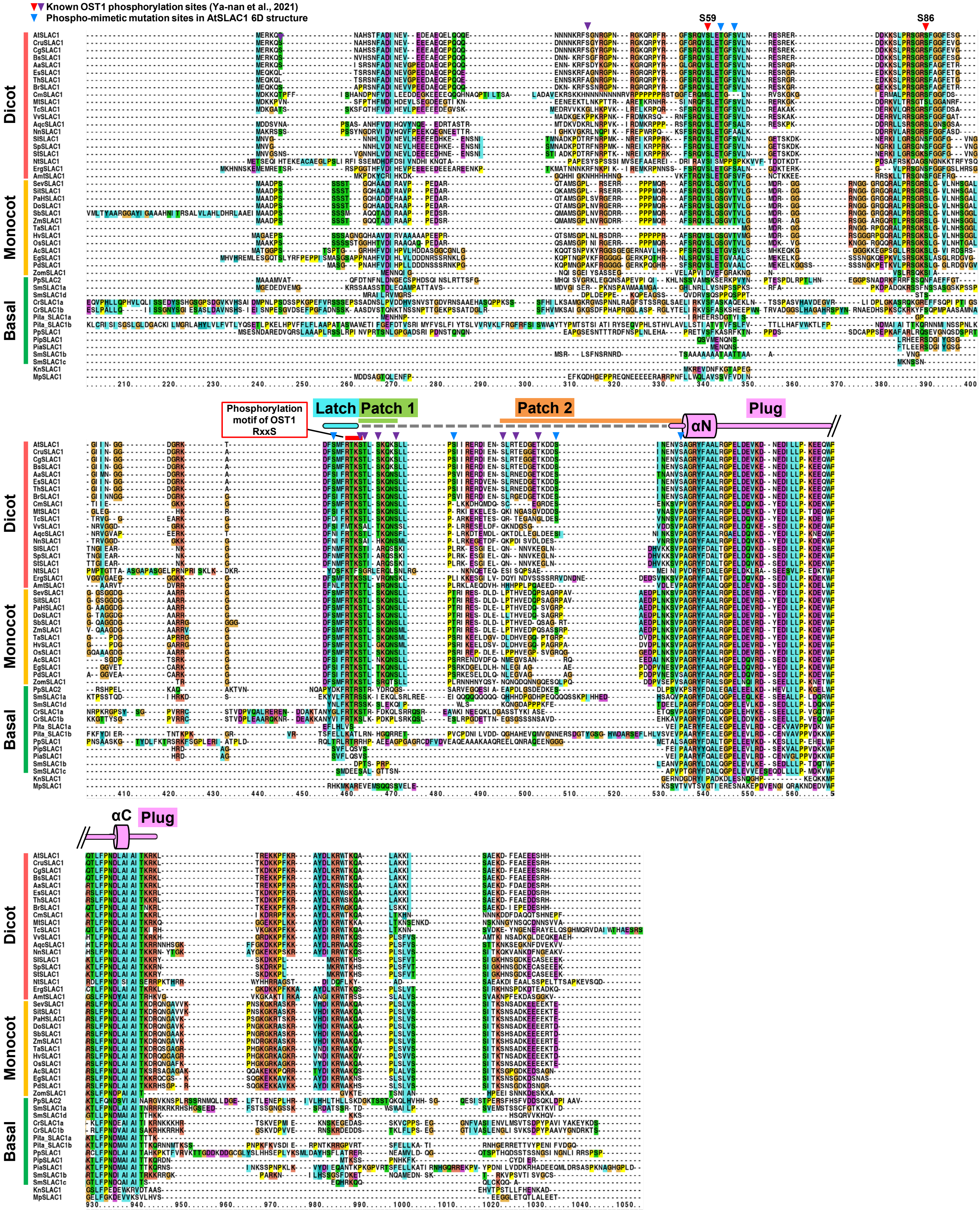

Supplementary Figure 16. Multiple sequence alignment of N- and C-terminal domains of SLAC1 orthologues.

Multiple sequence alignments of orthologous SLAC1 sequences from dicot, monocot and basal plants and two ancient SLAC1 orthologues from a charophyte *Klebsormidium nitens* (Kn) and a liverwort *Marchantia polymorpha* (Mp) based on a previous study[2]. Known phosphorylation sites (red and purple) and phospho-mimetic mutation sites (blue) are indicated by inverted-triangle. The sequence alignment was performed by ClustalX[3].

Supplementary Table 1. Cryo-EM data collection, refinement and validation statistics

	AtSLAC1 6D Closed (EMDB-34303) (PDB 8GW6)	AtSLAC1 6D Open (EMDB-34304) (PDB 8GW7)	AtSLAC1 8D Closed (EMDB-35904) (PDB 8J0J)	AtSLAC1 Open (EMDB-35920) (PDB 8J1E)
**Data collection and processing**				
Magnification	105,000 x	100,000 x	105,000 x	100,000 x
Voltage (kV)	300	200	300	200
Electron exposure (e^{-}/Å^2)	50	50	50	50
Defocus range (μm)	-0.7 ~ -1.9	-0.8 ~ -1.9	-0.7 ~ -1.9	-0.7 ~ -1.9
Pixel size (Å)	0.85	0.84	0.858	0.84
Symmetry imposed	C3	C3	C3	C3
Initial particle images (no.)	737,780	681,232	1,363,251	336,292
Final particle images (no.)	69,390	55,882	181,365	57,879
Map resolution (Å)	3.3	3.3	2.7	3.8
FSC threshold	0.143	0.143	0.143	0.143
Map resolution range (Å)	3.1 - 30	3.2 - 34	2.3 - 34	3.4 ~ 10.3
**Refinement**				
Initial model used (PDB code)	7WNQ	3M71	8GW6	8GW7
Model resolution (Å)	3.8	3.9	2.7	3.9
FSC threshold	0.5	0.5	0.5	0.5
Map sharpening *B* factor (Å^2)	151.4	142.8	110.9	164.3
Model composition				
Non-hydrogen atoms	9030	7524	9285	7590
Protein residues	1101	945	1131	954
Ligands	6	9	6	9
B factors (Å^2)				
Protein	59.70	86.29	9.41	51.96
Ligand	20.69	54.10	17.38	36.89
R.m.s. deviations				
Bond lengths (Å)	0.004	0.004	0.005	0.003
Bond angles (°)	0.539	0.683	0.573	0.640
Validation				
MolProbity score	1.84	1.3	1.60	1.45
Clash score	20.00	5.54	9.99	8.34
Poor rotamers (%)	0.65	0.39	1.25	0.00
Ramachandran plot				
Favored (%)	97.81	98.71	98.39	98.09
Allowed (%)	2.19	1.29	1.61	1.91
Disallowed (%)	0.00	0.00	0.00	0.00

Supplementary References

1. Krissinel, E. & Henrick, K. Inference of macromolecular assemblies from crystalline state. *J. Mol. Biol.* **372**, 774-797 (2007). https://doi.org/10.1016/j.jmb.2007.05.022

2. Schafer, N. *et al.* A tandem amino acid residue motif in guard cell SLAC1 anion channel of grasses allows for the control of stomatal aperture by nitrate. *Curr. Biol.* **28**, 1370-1379 (2018). https://doi.org:10.1016/j.cub.2018.03.027

3. Larkin, M. A. *et al.* Clustal W and Clustal X version 2.0. *Bioinformatics* **23**, 2947-2948 (2007). https://doi.org:10.1093/bioinformatics/btm404

Uncropped images of blots and gels

Supplementary Figure 2a.

HA

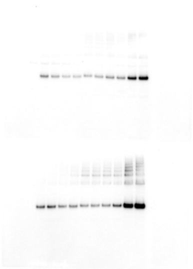

Actin

TfR

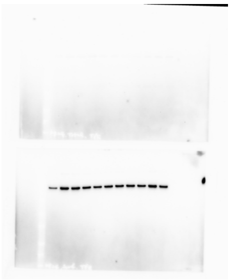

Supplementary Figure 2b.

HA

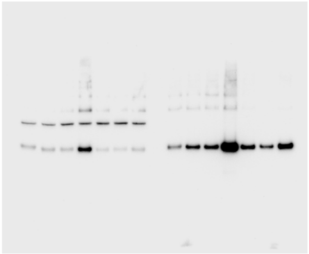

Actin

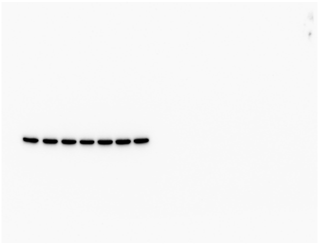

TfR

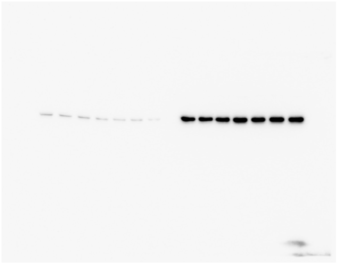

Supplementary Figure 2c.

HA

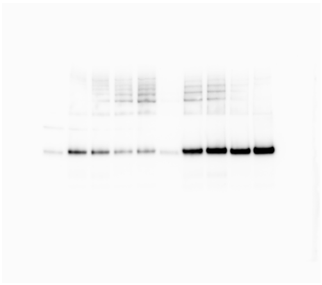

Actin

TfR

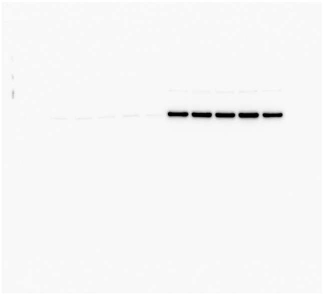

Supplementary Figure 3a.

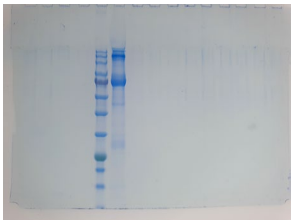

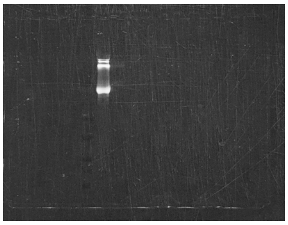

Supplementary Figure 4a.

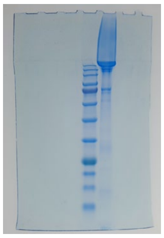

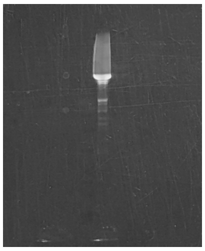

Supplementary Figure 5a.

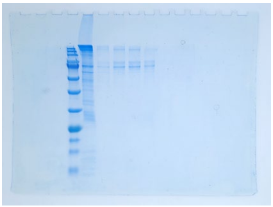

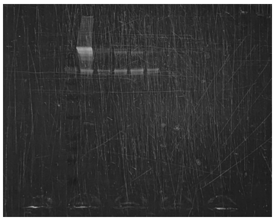

Supplementary Figure 6a.

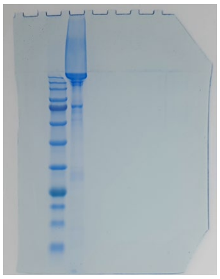

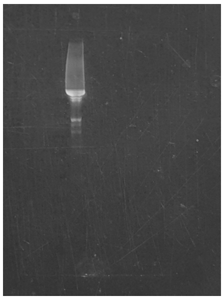

Supplement: Supplementary file 1 — Supplementary Information [file 41467_2023_43193_MOESM1_ESM.pdf]
