# Supplementary material for: Cryo-EM structures of the plant anion channel SLAC1 from Arabidopsis thaliana suggest a combined activation model
Source: Nat Commun. 2023 Nov 14;14:7345. doi: 10.1038/s41467-023-43193-3 (PMC10645844; doi:10.1038/s41467-023-43193-3)
Supplement: Supplementary file 3 — Reporting Summary [file 41467_2023_43193_MOESM3_ESM.pdf]

nature portfolio

Corresponding author(s): Sangho Lee

Last updated by author(s): Oct 23, 2023

Reporting Summary

Nature Portfolio wishes to improve the reproducibility of the work that we publish. This form provides structure for consistency and transparency in reporting. For further information on Nature Portfolio policies, see our Editorial Policies and the Editorial Policy Checklist.

Statistics

For all statistical analyses, confirm that the following items are present in the figure legend, table legend, main text, or Methods section.

n/a	Confirmed	
☐	☒	The exact sample size (*n*) for each experimental group/condition, given as a discrete number and unit of measurement
☐	☒	A statement on whether measurements were taken from distinct samples or whether the same sample was measured repeatedly
☐	☒	The statistical test(s) used AND whether they are one- or two-sided<br>*Only common tests should be described solely by name; describe more complex techniques in the Methods section.*
☒	☐	A description of all covariates tested
☒	☐	A description of any assumptions or corrections, such as tests of normality and adjustment for multiple comparisons
☐	☒	A full description of the statistical parameters including central tendency (e.g. means) or other basic estimates (e.g. regression coefficient) AND variation (e.g. standard deviation) or associated estimates of uncertainty (e.g. confidence intervals)
☐	☒	For null hypothesis testing, the test statistic (e.g. *F*, *t*, *r*) with confidence intervals, effect sizes, degrees of freedom and *P* value noted<br>*Give P values as exact values whenever suitable.*
☒	☐	For Bayesian analysis, information on the choice of priors and Markov chain Monte Carlo settings
☒	☐	For hierarchical and complex designs, identification of the appropriate level for tests and full reporting of outcomes
☒	☐	Estimates of effect sizes (e.g. Cohen's *d*, Pearson's *r*), indicating how they were calculated

Our web collection on statistics for biologists contains articles on many of the points above.

Software and code

Policy information about availability of computer code

Data collection	Cryo-EM data were collected using EPU software (Thermo Fisher Scientific)<br>Electrophysiological data were collected using pCLAMP 11 software (Molecular Devices)
Data analysis	Cryosparc v3.0 - 4.0<br>COOT v0.9.8.1<br>Phenix v1.20<br>PyMOL v2.5.0<br>UCSF Chimera v1.1<br>UCSF ChimeraX v1.6.1<br>ClustalX v2.1<br>MOLE v2.5<br>PDBePISA server (https://www.ebi.ac.uk/pdbe/pisa/pistart.html)<br>WebLogo server (https://weblogo.berkeley.edu/logo.cgi)<br>GraphPad Prism 8.4.3

For manuscripts utilizing custom algorithms or software that are central to the research but not yet described in published literature, software must be made available to editors and reviewers. We strongly encourage code deposition in a community repository (e.g. GitHub). See the Nature Portfolio guidelines for submitting code & software for further information.

Data

Policy information about availability of data

All manuscripts must include a data availability statement. This statement should provide the following information, where applicable:

- Accession codes, unique identifiers, or web links for publicly available datasets
- A description of any restrictions on data availability
- For clinical datasets or third party data, please ensure that the statement adheres to our policy

The structure coordinates have been deposited in the Protein Data Bank under accession codes 8GW6 for the AtSLAC1 6D closed structure, 8GW7 for AtSLAC1 6D open structure, 8J0J for AtSLAC1 8D closed structure and 8J1E for AtSLAC1 WT open structure. The corresponding cryo-EM density maps with local-filtering, half-maps and masks have been deposited in the Electron Microscopy Data Bank under accession codes EMDB-34303 for the AtSLAC1 6D closed structure, EMDB-34304 for AtSLAC1 6D open structure, EMDB-35904 for the AtSLAC1 8D closed structure and EMDB-35920 for AtSLAC1 WT open structure. The source data are provided with this paper.

Research involving human participants, their data, or biological material

Policy information about studies with human participants or human data. See also policy information about sex, gender (identity/presentation), and sexual orientation and race, ethnicity and racism.

Reporting on sex and gender	N/A
Reporting on race, ethnicity, or other socially relevant groupings	N/A
Population characteristics	N/A
Recruitment	N/A
Ethics oversight	N/A

Note that full information on the approval of the study protocol must also be provided in the manuscript.

Field-specific reporting

Please select the one below that is the best fit for your research. If you are not sure, read the appropriate sections before making your selection.

☒ Life sciences ☐ Behavioural & social sciences ☐ Ecological, evolutionary & environmental sciences

For a reference copy of the document with all sections, see nature.com/documents/nr-reporting-summary-flat.pdf

Life sciences study design

All studies must disclose on these points even when the disclosure is negative.

Sample size	All Experiments were repeated as much as possible, at least three times.
Data exclusions	No data were excluded from electrophysiological data. In cryo-EM, homogeneous particles with high resolution were only used to reconstruct map in refinement as described in data processing workflow.
Replication	All electrophysiological experiments were repeated at least three times, and indicated in figures
Randomization	Randomizations of experiments was not relevant to this study, and all measurements were independent from others
Blinding	Blinding experiments were not relevant to this study

Reporting for specific materials, systems and methods

We require information from authors about some types of materials, experimental systems and methods used in many studies. Here, indicate whether each material, system or method listed is relevant to your study. If you are not sure if a list item applies to your research, read the appropriate section before selecting a response.

Materials & experimental systems

n/a	Involved in the study
☒	☐ Antibodies
☐	☒ Eukaryotic cell lines
☒	☐ Palaeontology and archaeology
☒	☐ Animals and other organisms
☒	☐ Clinical data
☒	☐ Dual use research of concern
☒	☐ Plants

Methods

n/a	Involved in the study
☒	☐ ChIP-seq
☒	☐ Flow cytometry
☒	☐ MRI-based neuroimaging

Eukaryotic cell lines

Policy information about cell lines and Sex and Gender in Research

Cell line source(s)	Sf9 (Gibco), HEK293T (ATCC)
Authentication	Cell lines were authenticated from the distributors as described above.
Mycoplasma contamination	Cell lines were not tested for mycoplasma contamination.
Commonly misidentified lines (See ICLAC register)	N/A
